# Supplementary material for: Block copolymer derived uniform mesopores enable ultrafast electron and ion transport at high mass loadings
Source: Nat Commun. 2019 Feb 8;10:675. doi: 10.1038/s41467-019-08644-w (PMC6368586; doi:10.1038/s41467-019-08644-w)
Supplement: Supplementary file 1 — Supplementary Information [file 41467_2019_8644_MOESM1_ESM.pdf]

Supplementary Information

Block Copolymer Derived Uniform Mesopores Enable Ultrafast Electron and Ion Transport at High Mass Loadings

Liu *et al.*

1. Supplementary Methods

1.1 Mass Loadings

The mass loading (m_s, in mg cm^{-2}) of MnO_2 on the carbon fibers was determined using the mass difference (in mg) before and after the deposition of MnO_2 ($m_{\text{after}} - m_{\text{before}}$). For self-limiting redox deposition, the mass loadings were calculated according to the stoichiometric relationship of 3C ~ $4MnO_2$ ~ ($m_{\text{after}} - m_{\text{before}}$) using the equation below.

$$m_s = \frac{(m_{\text{after}} - m_{\text{before}}) \times (4\text{M}_{\text{MnO2}})}{\Delta\text{M} \times S_{\text{geo}}} \quad (1)$$

where M_{MnO2} is the molar mass of MnO_2 (=86.9 g mol^{-1}), S_{geo} is the geometric area (in cm^2) of the carbon mat used for deposition, and $\Delta\text{M} = 4\text{M}_{\text{MnO2}} - 3\text{M}_{\text{C}}$ is the molecular mass difference (in g mol^{-1}) between 4 mol of MnO_2 and 3 mol of carbon, according to the following reaction:

$$4\text{KMnO}_4 + 3\text{C} + \text{H}_2\text{O} \rightarrow 4\text{MnO}_2 + \text{K}_2\text{CO}_3 + 2\text{KHCO}_3$$

For electrochemical deposition, the mass loadings (in mg cm^{-2}) of MnO_2 were calculated based on the mass difference before and after the electrodeposition:

$$m_s = \frac{m_{\text{after}} - m_{\text{before}}}{S_{\text{geo}}} \quad (2)$$

1.2 Capacitances

The gravimetric capacitance (C_{m}, in F g^{-1}) of a single electrode (in a symmetric two-electrode testing configuration) is calculated using CV curves:

$$C_m = \frac{2C_{device}}{m_{device}/2} = \frac{4}{2(U_H - U_L)v} \int_{U_L}^{U_H} I_m dV \quad (3)$$

where C_{device} is the measured capacitance of the device (in F), m_{device} is the total mass of the two electrodes (in g), I_m is the current density (in A g^{-1}), v is the scan rate (in V s^{-1}), and V_H and V_L are the upper and lower limit of the potential window (both in V), respectively. The current was normalized to the total mass of the active materials in both positive and negative electrodes.

The geometric-areal normalized capacitance ($C_{\text{s,geo}}$, in mF cm^{-2}), BET-areal normalized capacitance ($C_{\text{s,BET}}$, in μF cm^{-2}) and volumetric capacitance (C_{V}, in F cm^{-3}) of a single electrode are derived from C_{m} as follows:

$$C_{s,\text{geo}} = C_m \times m_s \quad (4)$$

$$C_{s,\mathrm{BET}} = \frac{C_m}{S_{\mathrm{BET}}} (\text{in F m}^{-2}) = \frac{C_m}{S_{\mathrm{BET}}} \times 100\ (\text{in µF cm}^{-2}) \quad (5)$$

$$C_V = C_m \times m_V \quad (6)$$

where m_s, S_{BET}, and m_V represent areal mass loading (in mg cm^{-2}), BET specific surface area (in m^2 g^{-1}) and electrode packing density (in g cm^{-3}), respectively.

1.3 Energy Density and Power Density

Gravimetric power density (P_m, W kg^{-1}) and gravimetric energy density (E_m, Wh kg^{-1}) are evaluated in a two-electrode symmetric configuration and based on the total mass of the two electrodes.

$$E_m = \frac{(C_m/4) \times (U_H - U_L)^2}{2} (\text{in W s g}^{-1}) = \frac{(C_m/4) \times (U_H - U_L)^2}{2} \times \frac{1000}{3600} (\text{in Wh kg}^{-1}) \quad (7)$$

$$P_m = \frac{3600 E_m}{t_{\mathrm{discharge}}} = \frac{3600 E_m v}{U_H - U_L} \quad (8)$$

Where $C_m/4$ represents the device capacitance of a two-electrode symmetric pseudocapacitor device (in F g^{-1}), $t_{\mathrm{discharge}}$ is the discharge time (in s) and the coefficient 3600 is the conversion factor from hour to second (1 h = 3600 s). Other parameters follow the same definition as defined.

1.4 Capacitance Differentiation (The Dunn's Method)

We used the Dunn's method to quantify the capacitance contribution from fast-kinetic processes (including electrical double layer capacitive processes and fast redox reactions) and slow-kinetic processes (redox reactions that are diffusion-controlled).

First, the current density at a fixed potential and a scan rate, *i*, was extracted from the CV curves. According to Wang *et al.*,[1] the current density, *i,* is a function of the scan rate, v, and can be expressed as the sum of two terms v:

$$i(v) = \mathrm{k}_1 v + \mathrm{k}_2 v^{0.5} \quad (9)$$

where k_1 and k_2 are constants. The first term $k_1 v$ equals the current density contributed from fast-kinetic processes and the second term $k_2 v^{0.5}$ is the current density associated with slow-kinetic (or diffusion-controlled) processes. By dividing $v^{0.5}$ on both sides of the equation, it yields:

$$i\, v^{-0.5} = \mathrm{k}_1 v^{0.5} + \mathrm{k}_2 \quad (10)$$

Therefore, $i\,v^{-0.5}$ and $v^{0.5}$ are expected to have a linear relationship. The slope equals k_1 and the y-intercept equals k_2. By repeating the above steps for other potentials and scan rates, the capacitance contribution from the fast-kinetic and slow-kinetic processes can be mapped out.

1.5 *b*-value Analysis

The *b*-value analysis was performed to evaluate the charge-storage kinetics of the electrodes by cyclic voltammetry. According to Augustyn *et al.*,[2] the current densities at different scan rates and a fixed potential obey the following power-law relationship:

$$i(v) = \mathrm{k}v^{b} \tag{11}$$

where k is a pre-exponential constant and *b* is a real number between 0.5 and 1.0. When *b* equals 0.5, the charge-storage processes are sluggish due to the slow ion diffusion in the electrode. For instance, most battery electrodes store charges via slow solid-state ion diffusion and thus their *b* values typically approximate to 0.5. When *b* equals 1.0, the charge-storage processes are rapid and are not diffusion limited. For supercapacitor electrodes that store charges via surface reaction/sorption, solid-state diffusion is not involved and thus the *b* values are expected to be close to 1.0. For pseudocapacitor electrodes that involve ion diffusion across a thick layer of transition metal oxides, the *b* values deviate from 1.0. Typically, large deviations of the *b* values from one signify slow electron conduction and/or ion diffusion.

To obtain the *b* value, one can take logarithm on both sides of Supplementary Equation 11 and convert it to the following:

$$\log_{10}i = b\log_{10}v + C \tag{12}$$

where C is a constant that equals $\log_{10}$k. Based on Supplementary Equation 12, a linear relationship shall be observed between *i* and *v* in a logarithmic scale. The *b* value is the slope of the best linear fitting line.

1.6 SAX characterizations

The center-to-center pore-spacing, *d* (in nm), was estimated based on the SAXS spectra:

$$d = \frac{2\pi}{|\mathbf{q}|} \tag{13}$$

where $|\mathbf{q}|$ is the magnitude of characteristic scattering vector (in nm^{-1}).

2. Supplementary Figures

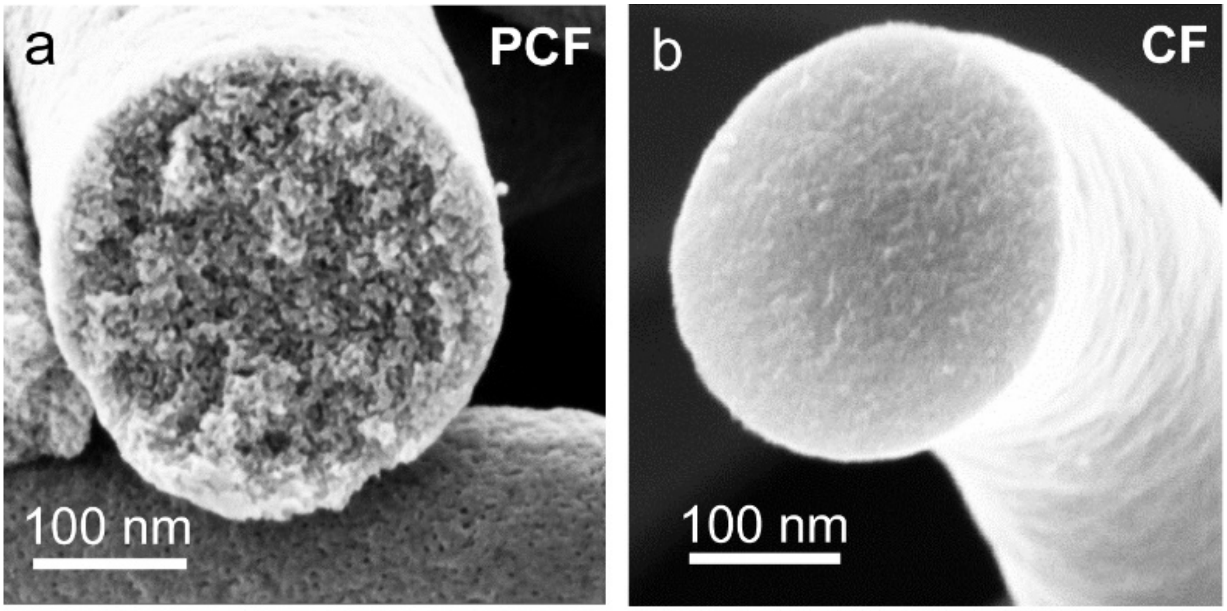

Supplementary Figure 1 | Representative cross-sectional SEM images of a fiber of (a) PCFs derived from PAN-*b*-PMMA and (b) conventional CFs. The PCFs show a large number of uniformly distributed, randomly oriented, and interconnected mesopores.

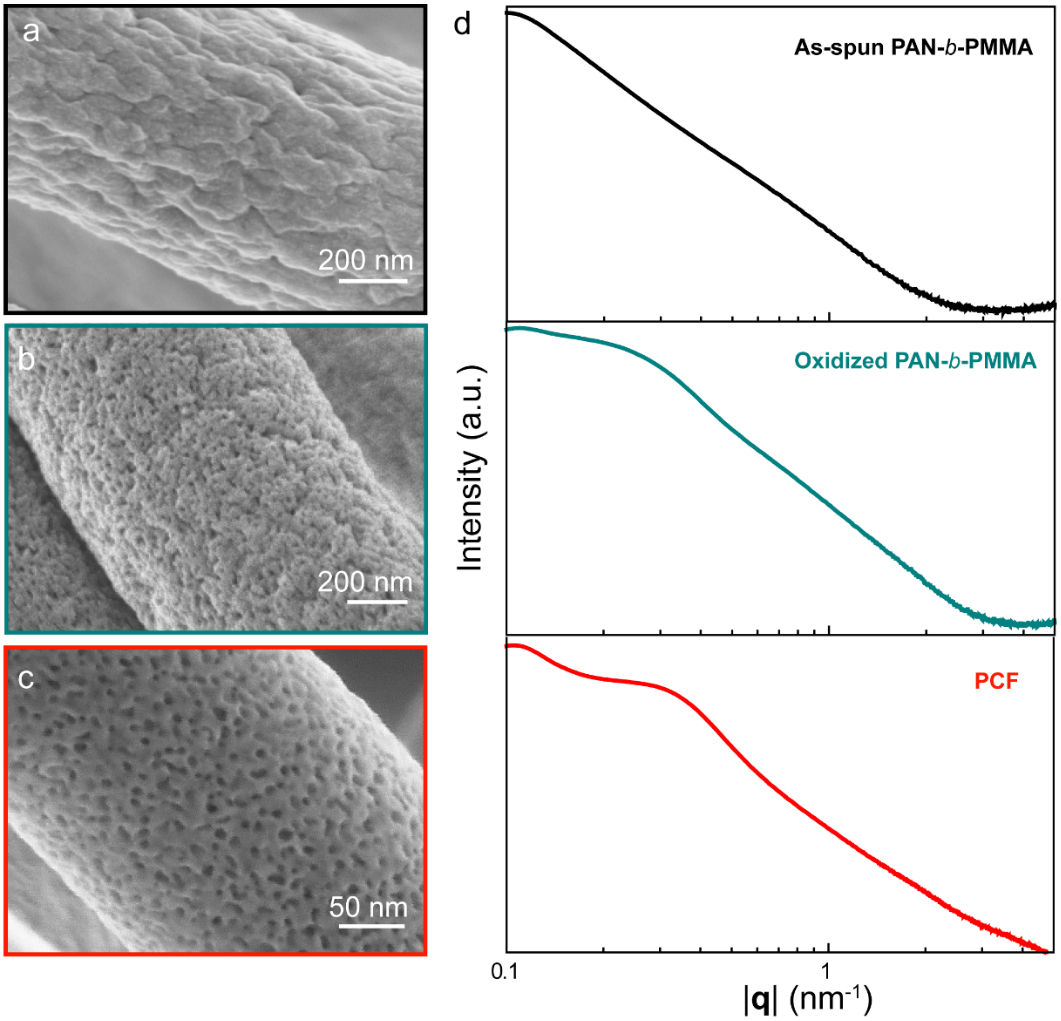

Supplementary Figure 2 | Morphologies and SAXS spectra. (a-c) SEM images of (a) as-spun PAN-*b*-PMMA, (b) oxidized PAN-*b*-PMMA and (c) PCF fibers. (d) SAXS spectra of as-spun PAN-*b*-PMMA, oxidized PAN-*b*-PMMA, and PCF fibers.

Under SEM, the surfaces of the as-spun PAN-*b*-PMMA fibers are wrinkled but devoid of any distinct feature (Supplementary Figure 2a), while those of the oxidized PAN-*b*-PMMA and PCFs contain dark domains (Supplementary Figures 2b and 2c). Because PMMA degrades at ~320 ^{o}C-430 °C (ref.[3]), the bright and dark domains in the oxidized PAN-*b*-PMMA are PAN and PMMA, respectively. Correspondingly, the bright and dark regions in PCFs are PAN-derived carbon and pores, respectively.

The SAXS spectra corroborate the SEM images. The SAXS spectrum of PAN-*b*-PMMA is featureless, confirming that PAN-*b*-PMMA lacks well-defined microstructures. After oxidation, a broad Bragg peak appears at ~0.196 nm^{-1}, corresponding to an average center-to-center pore-spacing of 32.0 nm. After pyrolysis at 1200 °C, the peak shifts to ~0.244 nm^{-1} and the average center-to-center pore-spacing reduces to 25.7 nm. The reduction in the pore-spacing is resulted from the shrink of the materials after pyrolysis. The broad SAXS peaks are due to the cross-linking of PAN, which prevents PAN-*b*-PMMA from forming well-defined cylindrical nanostructures.

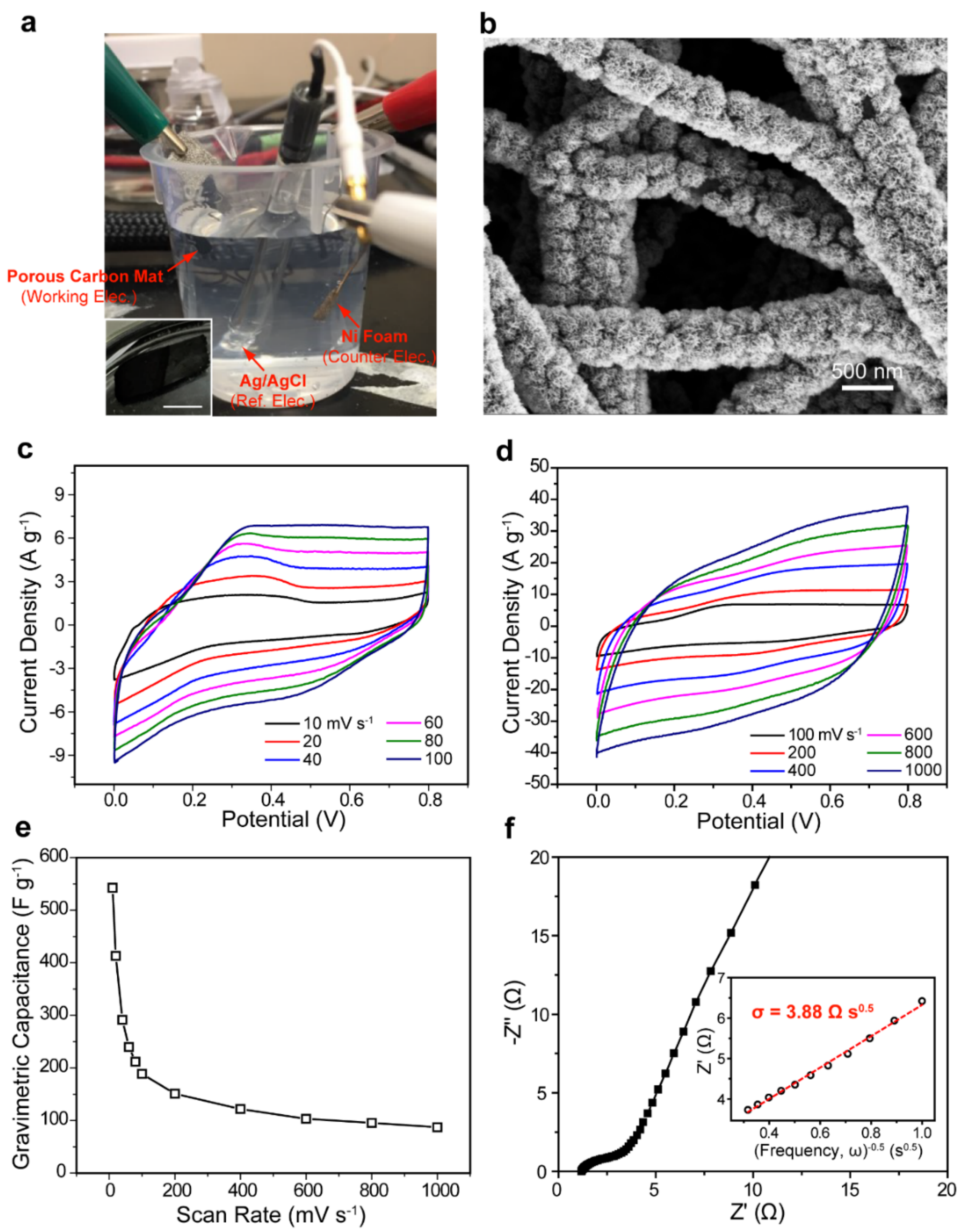

Supplementary Figure 3 | PCFs with electrodeposited MnO_2 nanoflowers. (a) A three-electrode setup for the electrodeposition of MnO_2 on PCFs. (Inset) A digital photograph of a piece of electrochemical deposited PCF mat. Scale bar, 1 cm. (b) Representative SEM image of electrodeposited MnO_2 on PCFs. (c and d) Cyclic voltammograms (CVs) at scan rates of (c) 10-100 mV s^{-1} and (d) 100-1000 mV s^{-1}. All CVs have a non-rectangular shape. (e) The rate capability and (f) Nyquist plot of the electrodeposited MnO_2 on PCFs. (Inset) Z' against the reciprocal of the square root of frequency, ω. The best linear fitting line (red) shows a diffusion

resistance, σ, of 3.88 Ω $s^{-0.5}$, much higher than that of MnO_2 on PCFs via redox reaction deposition.

In comparison with the solution-based redox reaction, we carried out the electrochemical deposition of MnO_2 on PCFs. The deposition was performed on a three-electrode setup (Supplementary Figure 3a) using cyclic voltammetry (0.01 mV s^{-1}, 15 cycles between 0 and 1.0 V). Porous carbon fiber mats served as the working electrodes, Ag/AgCl (in a saturated KCl aqueous solution) as the reference electrode, and a piece of nickel foam as the counter electrode. The aqueous electrodeposition solution contained 0.5 M LiCl and 0.1 M $Mn(CH_3COO)_2$. After electrochemical deposition, the MnO_2-deposited carbon fiber mat appeared darker than the un-deposited part (Supplementary Figure 3a inset). The electrochemical deposited MnO_2 were nanoflowers on the carbon fibers (Supplementary Figure 3b). Uniform clusters of MnO_2 were visible on each carbon fiber. The mass loading of MnO_2 was 4.2 mg cm^{-2}. Taking the mass of the carbon fiber mat into consideration, the total mass loading of the entire electrode reached 8.0 mg cm^{-2}.

The electrochemical performance of the electrochemical deposited MnO_2 on PCFs was evaluated by cyclic voltammetry. Supplementary Figures 3c and 3d present the collected CVs at various scan rates. The gravimetric capacitances calculated from the CVs (Supplementary Figure 3c and d) were summarized (Supplementary Figure 3e) to show the rate capability performance. At 10 mV s^{-1}, the electrochemical deposited MnO_2@PCFs obtained a gravimetric capacitance of 542.5 F g^{-1}, which was higher than that of the redox reaction deposited PCF@MnO_2-1h (481.3 F g^{-1}) and PCF@MnO_2-2h (461.9 F g^{-1}) at the identical scan rate (Figure 6c). It retained, however, only 87.1 F g^{-1} (16% of the capacitance at 10 mV s^{-1}) at 1000 mV s^{-1}. This capacitance is significantly lower than that of the redox reaction deposited PCF@MnO_2-2h (231.7 F g^{-1}) at the

identical scan rate. The poor rate capability is ascribed to the high diffusion resistance of 3.88 Ω $s^{0.5}$ (Supplementary Figure 3f inset) compared to that of PCF@MnO_2-2h (1.68 Ω $s^{0.5}$, Figure 4b).

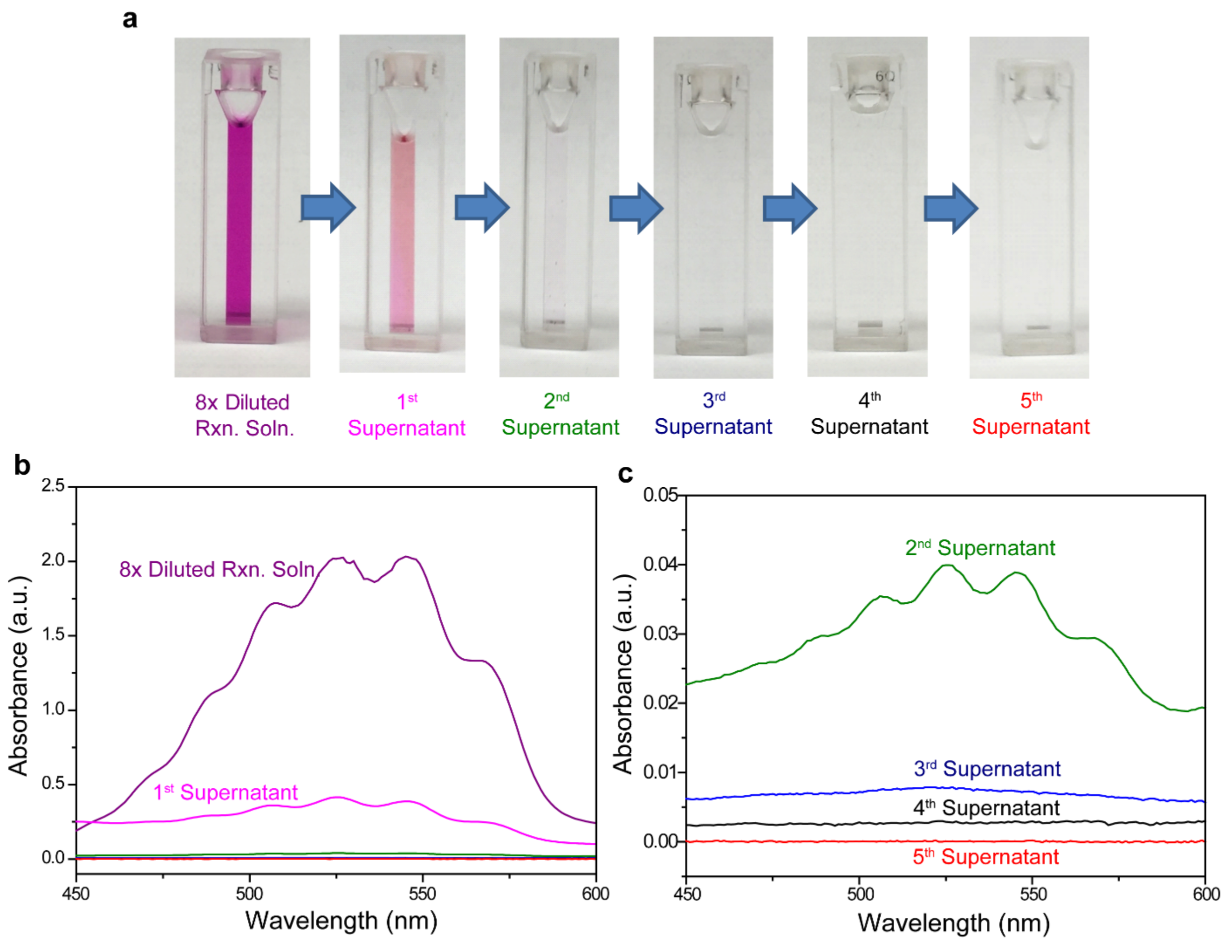

Supplementary Figure 4 | (a) Digital photographs and (b, c) UV-vis spectra of DI water that have been used to rinse the carbon fiber mats for various number of cycles. After rinsing, the DI water (denoted as supernatant) may contain $KMnO_4$ from the carbon fiber mat.

UV-vis spectroscopy was employed to monitor the concentration of residual $KMnO_4$ (from the MnO_2 redox deposition solution) in the supernatants after various number of rinsing cycles. The peaks between 450 and 600 nm are characteristic of $KMnO_4$, corresponding to the charge transfer between O and Mn. Supplementary Figure 4c is a magnified view of Supplementary Figure 4b, showing that the amount of $KMnO_4$ became undetectable after rinsing PCF@MnO_2 mats for five times.

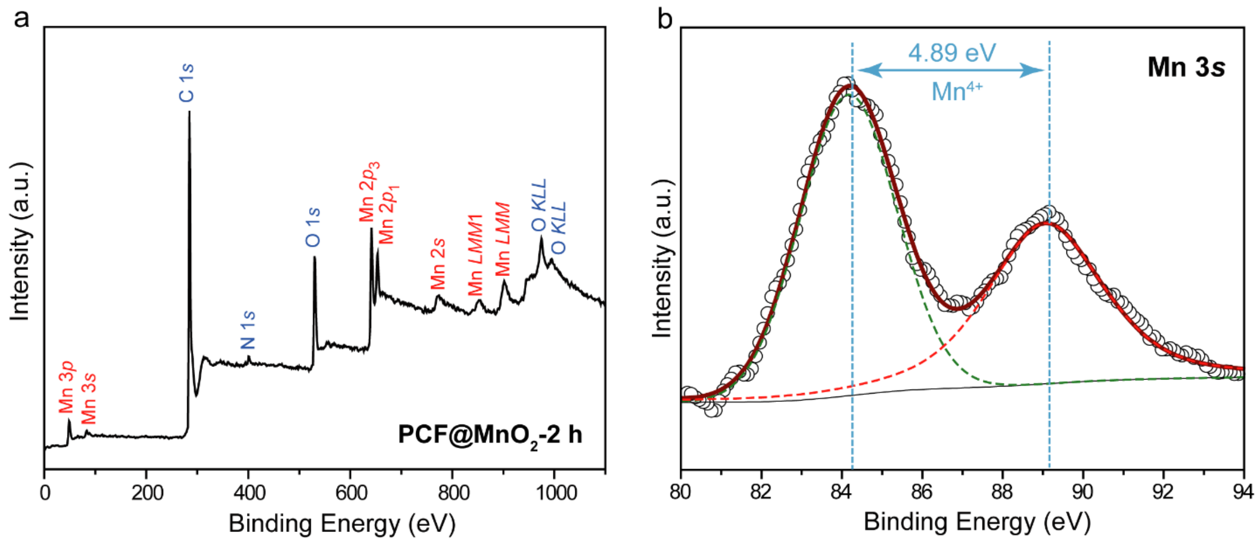

Supplementary Figure 5 | (a) XPS survey spectrum of PCF@MnO_2-2 h. The peaks associated with Mn are in red. (b) Mn 3*s* core-level XPS spectrum. The open circles are the experimental data. The solid and dashed curves represent the best fitting curves. The dotted blue lines highlight the peak position of the Mn 3*s* doublet. The peak separation confirms the valence state of Mn is +4 (MnO_2).

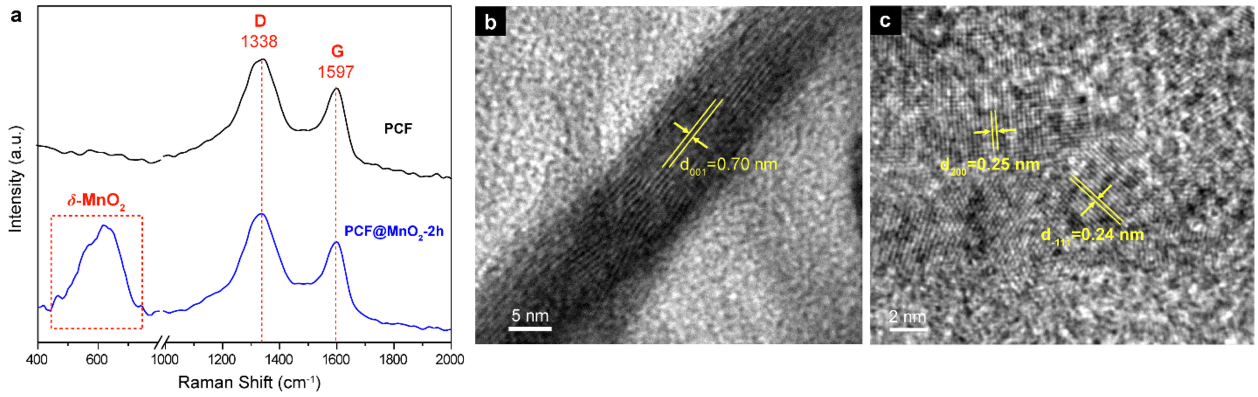

Supplementary Figure 6 | Raman and TEM Characterizations of MnO_2. (a) Raman spectra of PCF and PCF@MnO_2-2 h. The dashed box highlights the signature Raman peaks of δ-MnO_2. The dashed lines label the D and G peaks of PCF. (b and c) Lattice-resolved TEM images of δ-MnO_2 showing the signature lattice fringes.

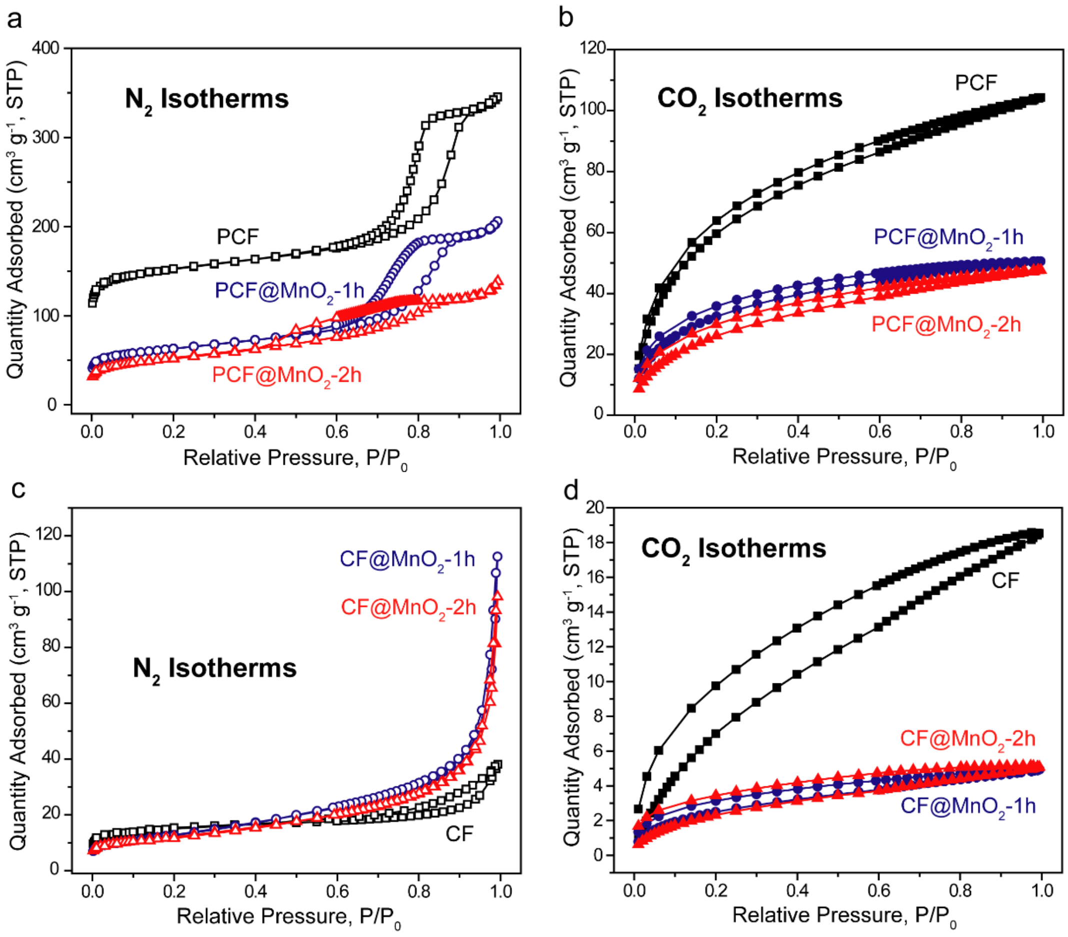

Supplementary Figure 7 | (a and c) Nitrogen (77 K) and (b and d) carbon dioxide (273 K) adsorption-desorption isotherms of (a and b) PCF- and (c and d) CF-based electrodes.

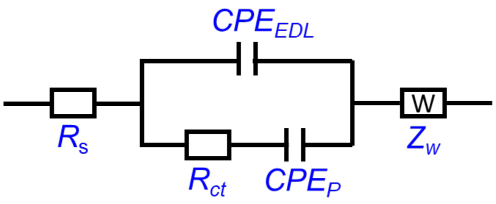

Supplementary Figure 8 | The equivalent electric circuit model used for fitting the Nyquist plots. R_s: combined series resistance; R_{ct}: charge transfer resistance; CPE_{EDL}: constant phase element representing the electrical double layer capacitance (EDLC); CPE_P: constant phase element representing the pseudocapacitance; Z_w: Warburg diffusion element.

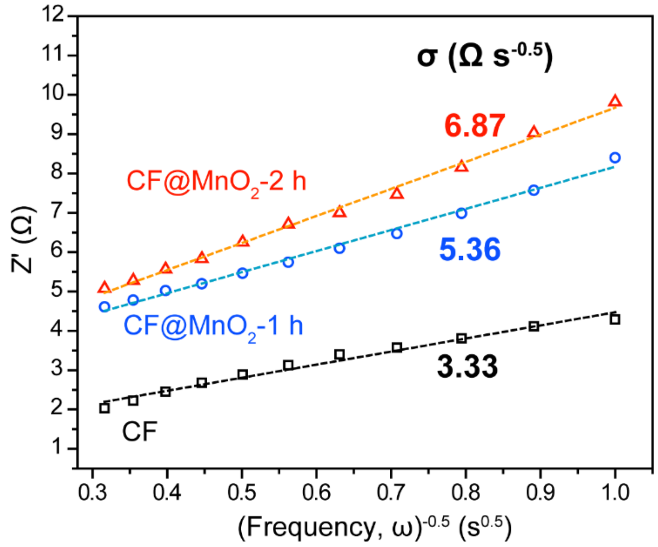

Supplementary Figure 9 | *Z'* *vs.* the reciprocal of the square root of frequency ($\omega^{-0.5}$) in the intermediate frequency range. The dashed lines are best fitting lines to calculate the diffusion resistance, σ. The σ of CF, CF@MnO_2-1h and CF@MnO_2-2h are 3.33, 5.36, and 6.87 Ω $s^{-0.5}$, respectively.

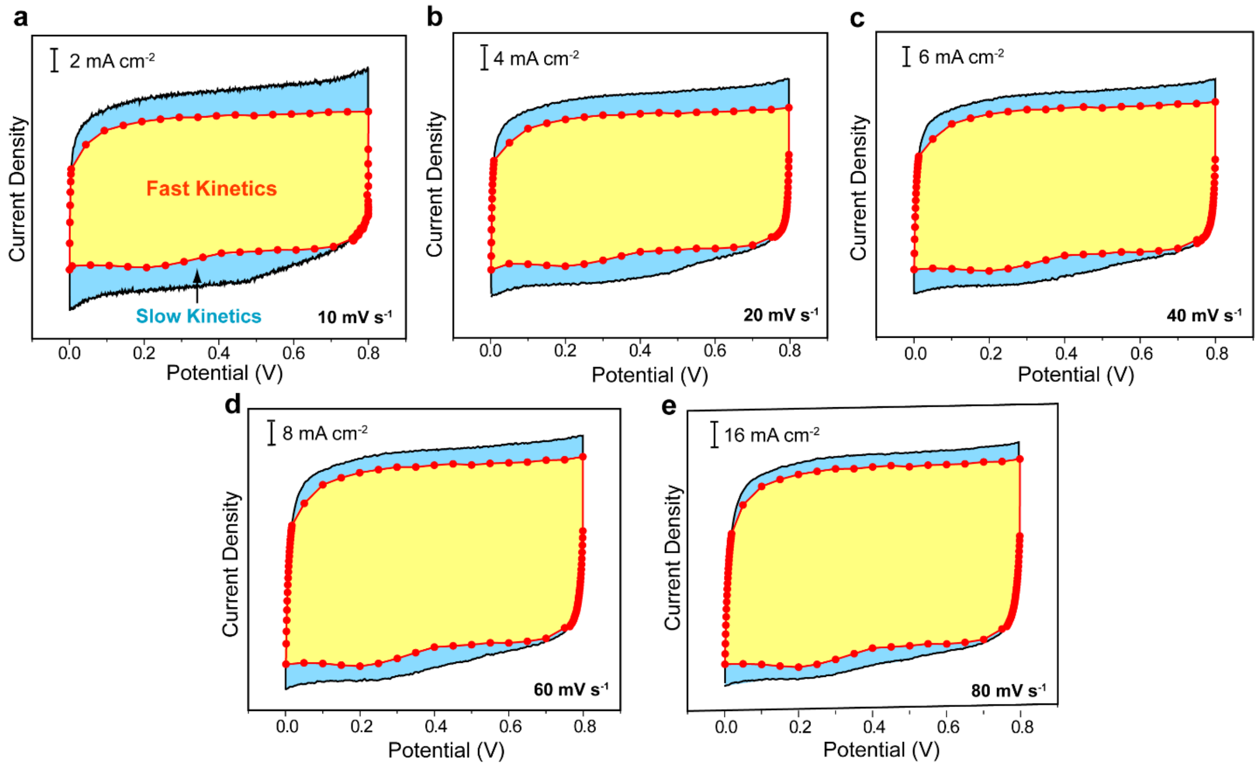

Supplementary Figure 10 | The decoupling of capacitance contribution from fast-kinetics processes (yellow) and slow-kinetics processes (blue). CVs are collected at various scan rates from 10 to 80 mV s^{-1}.

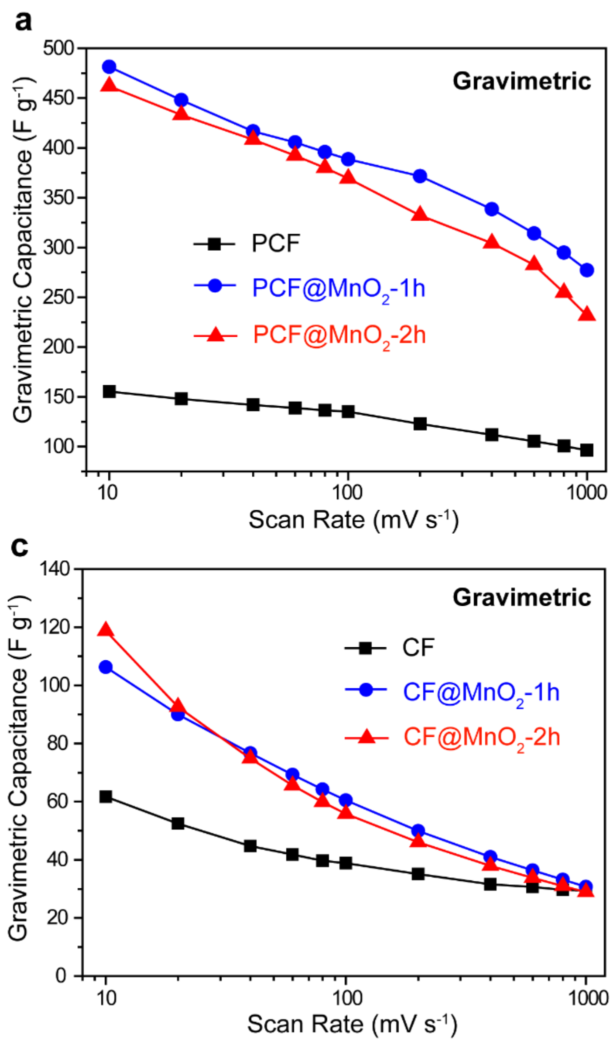

Supplementary Figure 11 | (a and c) Gravimetric and (b and d) geometric areal capacitances of all materials in (a and b) PCF- and (c and d) CF-based electrodes.

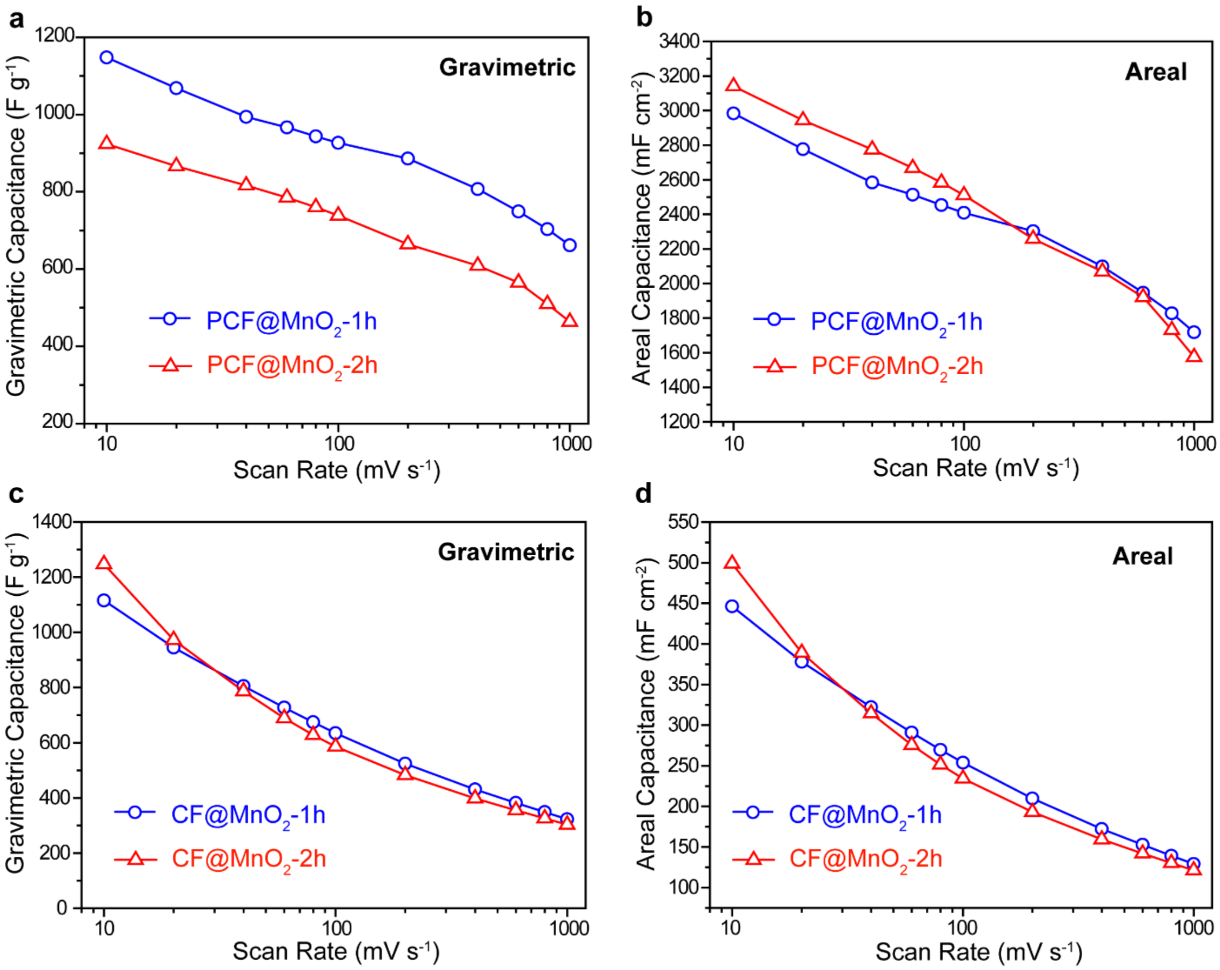

Supplementary Figure 12 | (a and c) Gravimetric and (b and d) geometric areal capacitances of MnO_2 in (a and b) PCF- and (c and d) CF-based electrodes.

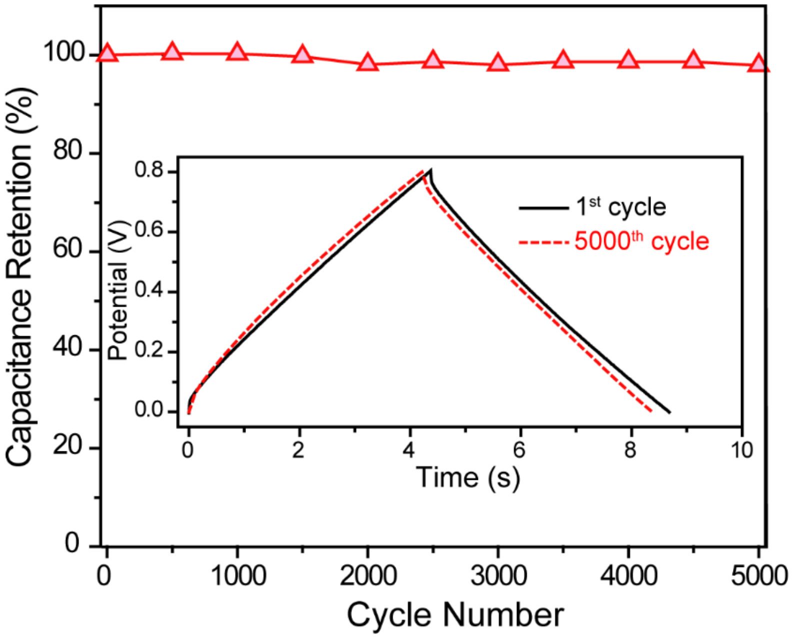

Supplementary Figure 13 | Cyclability performance. Charge-discharge cycling stability of PCF@MnO_2-2 h. (Inset) The galvanostatic charge-discharge profiles in the 1st and the 5000th cycles. The capacitance retained 98% of its initial value after the 5000 charge-discharge cycles.

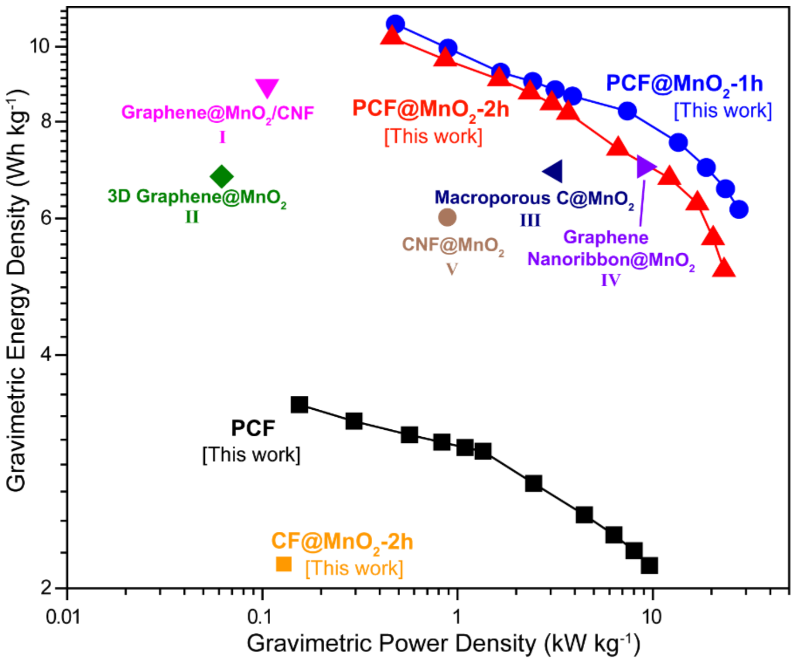

Supplementary Figure 14 | Ragone plots of PCF-based electrodes in comparison with CF and graphene-based electrodes in symmetric supercapacitors. References: I – Ref.4; II – Ref.5; III – Ref.6; IV – Ref.7; V– Ref.8. Solid lines are guides for eyes.

3. Supplementary Tables

Supplementary Table 1 | Surface area and porosity of CF- and PCF-based electrodes.

Sample	BET Surface Area (m^2 g^{-1})	Micropore Volume (cm^3 g^{-1})	Mesopore Volume (cm^3 g^{-1})	Macropore Volume (cm^3 g^{-1})
PCF	574.8±1.9	0.1440	0.8690	0.0067
PCF@MnO_2-1h	229.6±0.4	0.0582	0.2100	0.0063
PCF@MnO_2-2h	187.5±1.1	0.0494	0.1200	0.0062
CF	55.3±0.1	0. 0177	0. 0210	0.0064
CF@MnO_2-1h	43.4±0.3	0.0048	0.1293	0.0644
CF@MnO_2-2h	41.8±0.2	0.0048	0.1172	0.0557

Supplementary Table 2 | The electrochemical performance of representative MnO_2-based pseudocapacitive electrodes.

[The data listed in this table are mainly selected from two comprehensive review articles by Wang *et al.*[9] and Huang *et al.*[10] Previous reports without mass loading information are not included. To meet the requirement of practical applications, in general the mass loading of the total active materials (including carbon support and pseudocapacitive materials) must be >5 mg cm^{-2}.[11] This table by no means is exhaustive but represents the state-of-the-art for comparison.]

Electrode	Substrate [a]	Method [b]	Mass Loading (mg cm^{-2})	Electrolyte	Gravimetric Capacitance (F g^{-1}) [c]	Areal Capacitance (mF cm^{-2}) [c]	Rate Capability	Ref.
**Based on the mass of MnO_2**								
PCF@MnO_2-1h	PCF	RD	2.6	6 M KOH	1147.8	2984	57.6% [10-1000 mV s^{-1}]	This Work
PCF@MnO_2-2h	PCF	RD	3.4	6 M KOH	923.8	3141	50.2% [10-1000 mV s^{-1}]	This Work
CF@MnO_2-1h	CF	RD	0.4	6 M KOH	1115.6	447	28.9% [10-1000 mV s^{-1}]	This Work
CF@MnO_2-2h	CF	RD	0.4	6 M KOH	1246.9	499	24.3% [10-1000 mV s^{-1}]	This Work
MnO_2 Nanosheets	Graphene Foam/CNT	HT	6.2	0.5 M Na_2SO_4	~75	~465	~26.7% [10-100 mV s^{-1}]	12
MnO_2 Nanosheets	CVD-Graphene	ED	6.6	0.5 M Na_2SO_4	~90	~594	~16.7% [10-100 mV s^{-1}]	5
MnO_2 Nanosheet Films	CC	ED	7.5	5 M LiCl	173.3 [5 mV s^{-1}]	1300 [5 mV s^{-1}]	57.7% [5-100 mV s^{-1}]	13
MnO_2/PEDOT	Activated CNTs	ED	3.1	1 M Na_2SO_4	~365 [5 mV s^{-1}]	~1132 [5 mV s^{-1}]	68.4% [5-100 mV s^{-1}]	14
MnO_2 Nanoflowers	Biomass-derived Macroporous C	ED	5.8	1 M Na_2SO_4	~130	~754	61.5% [10-100 mV s^{-1}]	6

MnO_2 Nanoflowers	CF	ED	3.8	0.5 M Na_2SO_4	~70	~266	14.3% [10-100 mV s^{-1}]	15
MnO_2 Nanoflowers	CF	ED	8.3	0.5 M Na_2SO_4	337 [0.05 mV s^{-1}]	2800 [0.05 mV s^{-1}]	57.1% [0.05-0.8 mV s^{-1}]	15
MnO_2 Nanoflower Films	CNT	ED	6.5	1 M Na_2SO_4	80 [20 mV s^{-1}]	520 [20 mV s^{-1}]	31.3% [20-200 mV s^{-1}]	16
MnO_2 Nanowires	Ni-Coated Cu Dendrite	ED	1.8	0.5 M Na_2SO_4	268	482	50.4% [10-100 mV s^{-1}]	17
MnO_2 Nanowires	Ni-Coated Cu Dendrite	ED	0.4	0.5 M Na_2SO_4	1125 [5 mV s^{-1}]	394 [5 mV s^{-1}]	50.3% [5-200 mV s^{-1}]	17
Al-Doped MnO_2	Glass Piece	HT	~4.0	0.5 M Na_2SO_4	213 [0.1 A g^{-1}]	852 [0.1 A g^{-1}]	51.5% [0.1-5 A g^{-1}]	18
O-deficient MnO_2 Nanorods	Ni Foam	ED	0.5	0.5 M Na_2SO_4	306	153	53.5% [10-100 mV s^{-1}]	19
Porous MnO_2	Ni Foam	RD	5.0	1 M Na_2SO_4	245	1225	83.7% [10-20 mV s^{-1}]	20
MnO_2 Films	Graphene-Coated Ni Foam	ED	1.0	0.5 M Na_2SO_4	322	322	45.7% [10-200 mV s^{-1}]	21
MnO_2 Nanoflowers	CNT-Coated CF	ED	4.1	1 M Na_2SO_4	268	1099	54.5 [10-100 mV s^{-1}]	22
MnO_2 Films	CNT/Graphene	RD	2.0	1 M Na_2SO_4	330	660	69.7% [10-100 mV s^{-1}]	4
Ultrathin MnO_2 Nanosheets	CNT on Ni Foam	RD	3.5	1 M Na_2SO_4	306 [1 A g^{-1}]	1071 [1 A g^{-1}]	40.0% [1-10 A g^{-1}]	23
Hierarchical MnO_2 Films	CC	ED	10.0	1 M Na_2SO_4	304 [3 mA cm^{-2}]	3040 [3 mA cm^{-2}]	62.5% [3-30 mA cm^{-2}]	24

MnO_2 Nanoclusters	Graphene/Activated Carbon	ED	10.0	1 M Na_2SO_4	122 [1 mA cm^{-2}]	918 [1 mA cm^{-2}]	61.3% [1-3 mA cm^{-2}]	25
Self-Branched MnO_2 Nanowires	CC	RD	3.4	1 M LiCl	152	517	76.3% [10-20 mV s^{-1}]	26
MnO_2 Nanosheets	Wood-Derived Carbon	ED	45.0	1 M Na_2SO_4	176.8 [1 mA cm^{-2}]	7956 [1 mA cm^{-2}]	43.5% [1-30 mA cm^{-2}]	27
MnO_2 Films	CNT	ED	0.7	0.5 M Na_2SO_4	542 [0.5 A g^{-1}]	379 [0.5 A g^{-1}]	79.3% [0.5-10 A g^{-1}]	28
Porous Layered MnO_2	Ni Foam	HT	2.5	1 M Na_2SO_4	253 [0.1 A g^{-1}]	633 [0.1 A g^{-1}]	62% [0.1-10 A g^{-1}]	29
MnO_2 Nanorods	CF Paper	ED	1.5	0.5 M Na_2SO_4	362.5 [0.5 A g^{-1}]	544 [0.5 A g^{-1}]	44.1% [0.5-5 A g^{-1}]	30
MnO_2 Nanosheets	CNT on Graphene Aerogel	RD	2.5	1 M Na_2SO_4	~200	~500	~60.0% [10-100 mV s^{-1}]	31
Hexagonal MnO_2 Nanosheets	CNT-Coated Paper	RD	0.08	0.5 M Na_2SO_4	1035 [2 mV s^{-1}]	92 [2 mV s^{-1}]	24.6% [2-100 mV s^{-1}]	32
MnO_2 Nanoflowers	Laser-Scribed Graphene Sheet	ED	0.02	1 M Na_2SO_4	1145	20.6	22.2% [10-100 mV s^{-1}]	33
β-MnO_2@δ-MnO_2	Ni Foam	HT+RD	1.2	1 M Na_2SO_4	657	788	58.8% [0.25-32 A g^{-1}]	34
Porous Hydrated MnO_2 Films	CC	ED	3.7	5 M LiCl	243 [1 mA cm^{-2}]	900	73.0% [1-20 mA cm^{-2}]	35
Porous Hydrated MnO_2 Films	CC	ED	7.0	5 M LiCl	234 [1 mA cm^{-2}]	1638 [1 mA cm^{-2}]	69.0% [1-20 mA cm^{-2}]	35
Porous MnO_2 Films	Nanoporous Au Foil	ED	0.09	2 M Li_2SO_4	~750	~68	~65.3% [10-100 mV s^{-1}]	36

MnO_2 Nanoflower	CF	ED	3.1	1 M Na_2SO_4	170 [3 mA cm^{-2}]	526	~62.4% [3-20 mA cm^{-2}]	37
MnO_2 Nanosheets	Graphenated CNT	ED	2.3	1 M Na_2SO_4	640 [1 mA cm^{-2}]	1472 [1 mA cm^{-2}]	23.4% [1-10 mA cm^{-2}]	38
MnO_2 Nanosheets	Ni Nanowire	ED	3.5	0.5 M Na_2SO_4	214 [1 mV s^{-1}]	749 [1 mV s^{-1}]	-	39
MnO_2 Nanosheets	Ni Nanowire	ED	0.3	0.5 M Na_2SO_4	680 [1 mV s^{-1}]	204 [1 mV s^{-1}]	-	39
α-MnO_2 Nanoflowers	Super-aligned CFs	ED	0.5	6 M KOH	630	315	-	40
MnO_2 Nanosheets	Exfoliated Graphite Foil	ED	0.2	3 M KCl	1061 [0.23 mA cm^{-2}]	244 [0.23 mA cm^{-2}]	44.7% [0.23-11.5 mA cm^{-2}]	41
MnO_2 Nanotubes	CNT	HT	4.0	0.5 M Na_2SO_4	51 [0.5 mA cm^{-2}]	205 [0.5 mA cm^{-2}]	-	42
MnO_2 Nanoparticles	Au Nanospike	ED	0.03	1 M Na_2SO_4	581	17	51.4% [10-500 mV s^{-1}]	43
PEDOT-Wrapped MnO_2 Nanopaticles	CNT/Au	RD	1.5	1 M Na_2SO_4	200 [5 mA cm^{-2}]	300 [5 mA cm^{-2}]	-	44
MnO_2 Nanoflowers	CNT Sponge	ED	0.04	1 M Na_2SO_4	~500	~20	88.0% [10-100 mV s^{-1}]	45
MnO_2 Films	Carbon Nanofoam	RD	7.5	1 M Na_2SO_4	200 [2 mV s^{-1}]	1500 [2 mV s^{-1}]	-	46
**Based on the total mass of electrodes**								
PCF@MnO_2-1h	PCF	RD	6.2	6 M KOH	481	2984	57.6% [10-1000 mV s^{-1}]	This Work

PCF@MnO_2-2h	PCF	RD	6.8	6 M KOH	462	3140	50.2% [10-1000 mV s^{-1}]	This Work
CF@MnO_2-1h	CF	RD	4.2	6 M KOH	106	446	28.9% [10-1000 mV s^{-1}]	This Work
CF@MnO_2-2h	CF	RD	4.2	6 M KOH	119	499	24.3% [10-1000 mV s^{-1}]	This Work
β-MnO_2@δ-MnO_2	Ni Foam	HT+RD	2.5	1 M Na_2SO_4	396 [0.25 A g^{-1}]	990 [0.25 A g^{-1}]	58.8% [0.25-32 A g^{-1}]	34
Mesoporous MnO_2 Nanoparticles	CNT	RD	1.0	1 M Na_2SO_4	260	260	57.7% [10-200 mV s^{-1}]	47
MnO_2 Nanofibers	CNT	TD	20	0.5 M Na_2SO_4	150	3000	40.7% [10-100 mV s^{-1}]	48
MnO_2 Nanoflowers	Laser-Scribed Graphene Sheet	ED	5.8	1 M Na_2SO_4	~78	~450	22.2% [10-100 mV s^{-1}]	33
MnO_2 Nanosheets	Graphene Hydrogel	RD	0.5	1 M Na_2SO_4	~350 [1 A g^{-1}]	175 [1 A g^{-1}]	~78.6% [1-10 A g^{-1}]	49
MnO_2 Nanowires	CF Network	ED	1.9	1 M Na_2SO_4	125 [0.5 A g^{-1}]	238 [0.5 A g^{-1}]	-	50
MnO_2 Nanosheets	Wood-Derived Carbon	ED	75	1 M Na_2SO_4	55 [1 mA cm^{-2}]	4155 [1 mA cm^{-2}]	44.0% [1-30 mA cm^{-2}]	27
MnO_2 Nanoparticles	rGO	RD	3.7	1 M Na_2SO_4	243 [0.05 A g^{-1}]	897 [0.05 A g^{-1}]	~35.7% [0.05-1 A g^{-1}]	51
MnO_2 Nanospheres	Carbon Nanospheres	TD	2.2	6 M KOH	459 [1 A g^{-1}]	1010 [1 A g^{-1}]	77.1% [1-20 A g^{-1}]	52
MnO_2 Nanotubes	CNT	HT	7.0	0.5 M Na_2SO_4	29 [0.5 mA cm^{-2}]	205 [0.5 mA cm^{-2}]	-	42

MnO_2 Nanoparticles	Activated Carbon-Coated CNT	RD	20	Saturated Na_2SO_4	~200	4000	30% [10-100 mV s^{-1}]	53
MnO_2 Films	Carbon Nanofoam	RD	13.6	1 M Na_2SO_4	110 [2 mV s^{-1}]	1500 [2 mV s^{-1}]	-	46
Birnessite Films	Porous Carbon Nanofoam	RD	16.7	0.1 M tetrabutylammonium perchlorate in acetonitrile	150 [2 mV s^{-1}]	2500 [2 mV s^{-1}]	-	54
Birnessite Films	Porous Carbon Nanofoam	RD	33.3	0.1 M tetrabutylammonium perchlorate in acetonitrile	150 [2 mV s^{-1}]	5000 [2 mV s^{-1}]	-	54
Birnessite Films	Porous Carbon Nanofoam	RD	50	0.1 M tetrabutylammonium perchlorate in acetonitrile	150 [2 mV s^{-1}]	7500 [2 mV s^{-1}]	-	54

Notes:

[a] CVD = Chemical Vapor Deposition; CC = Carbon Cloth; CNT = Carbon Nanotube; AC = Activated Carbon; CF = Carbon Fiber;

[b] ED = Electrodeposition; RD = Redox deposition with $KMnO_4$; HT = Hydrothermal reaction; TD = Thermal decomposition

[c] The listed gravimetric capacitances are values obtained at 10 mV s^{-1}, unless otherwise noted in the brackets.

Supplementary References

1. Wang J., Polleux J., Lim J., Dunn B. Pseudocapacitive contributions to electrochemical energy storage in TiO_2 (anatase) nanoparticles. *J. Phys. Chem. C* **111**, 14925-14931 (2007).

2. Augustyn V., *et al.* High-rate electrochemical energy storage through Li^+ intercalation pseudocapacitance. *Nat. Mater.* **12**, 518-522 (2013).

3. Zhou Z., Liu G. Controlling the pore size of mesoporous carbon thin films through thermal and solvent annealing. *Small* **13**, 1603107 (2017).

4. Cheng Y., Lu S., Zhang H., Varanasi C. V., Liu J. Synergistic effects from graphene and carbon nanotubes enable flexible and robust electrodes for high-performance supercapacitors. *Nano Lett.* **12**, 4206-4211 (2012).

5. He Y., *et al.* Freestanding three-dimensional graphene/MnO_2 composite networks as ultralight and flexible supercapacitor electrodes. *ACS Nano* **7**, 174-182 (2013).

6. Wang L., *et al.* Three-dimensional kenaf stem-derived porous carbon/MnO_2 for high-performance supercapacitors. *Electrochim. Acta* **135**, 380-387 (2014).

7. Liu M., Tjiu W. W., Pan J., Zhang C., Gao W., Liu T. One-step synthesis of graphene nanoribbon-MnO_2 hybrids and their all-solid-state asymmetric supercapacitors. *Nanoscale* **6**, 4233-4242 (2014).

8. Wang J.-G., Yang Y., Huang Z.-H., Kang F. A high-performance asymmetric supercapacitor based on carbon and carbon-MnO_2 nanofiber electrodes. *Carbon* **61**, 190-199 (2013).

9. Wang J.-G., Kang F., Wei B. Engineering of MnO_2-based nanocomposites for high-performance supercapacitors. *Prog. Mater. Sci.* **74**, 51-124 (2015).

10. Huang M., Li F., Dong F., Zhang Y. X., Zhang L. L. MnO_2-based nanostructures for high-performance supercapacitors. *J. Mater. Chem. A* **3**, 21380-21423 (2015).

11. Balducci A., Belanger D., Brousse T., Long J. W., Sugimoto W. A guideline for reporting performance metrics with electrochemical capacitors: from electrode materials to full devices. *J. Electrochem. Soc.* **164**, A1487-A1488 (2017).

12. Liu J., Zhang L., Wu H. B., Lin J., Shen Z., Lou X. W. High-performance flexible asymmetric supercapacitors based on a new graphene foam/carbon nanotube hybrid film. *Energy Environ. Sci.* **7**, 3709-3719 (2014).

13. Song Y., *et al.* Ostwald ripening improves rate capability of high mass loading manganese oxide for supercapacitors. *ACS Energy Lett.* **2**, 1752-1759 (2017).

14. Lv P., Feng Y. Y., Li Y., Feng W. Carbon fabric-aligned carbon nanotube/MnO_2/conducting polymers ternary composite electrodes with high utilization and mass loading of MnO_2 for super-capacitors. *J. Power Sources* **220**, 160-168 (2012).

15. Hu L., *et al.* Symmetrical MnO_2-carbon nanotube-textile nanostructures for wearable pseudocapacitors with high mass Loading. *ACS Nano* **5**, 8904-8913 (2011).

16. Narubayashi M., Chen Z., Hasegawa K., Noda S. 50–100 μm-thick pseudocapacitive electrodes of MnO_2 nanoparticles uniformly electrodeposited in carbon nanotube papers. *RSC Adv.* **6**, 41496-41505 (2016).

17. Sun Z., Firdoz S., Yap E. Y., Li L., Lu X. Hierarchically structured MnO_2 nanowires supported on hollow Ni dendrites for high-performance supercapacitors. *Nanoscale* **5**, 4379-4387 (2013).

18. Hu Z., *et al.* Al-doped α-MnO_2 for high mass-loading pseudocapacitor with excellent cycling stability. *Nano Energy* **11**, 226-234 (2015).

19. Zhai T., *et al.* Oxygen vacancies enhancing capacitive properties of MnO_2 nanorods for wearable asymmetric supercapacitors. *Nano Energy* **8**, 255-263 (2014).

20. Wan C., Yuan L., Shen H. Effects of electrode mass-loading on the electrochemical properties of porous MnO_2 for electrochemical supercapacitor. *Int. J. Electrochem. Sci.* **9**, 4024-4038 (2014).

21. Zhai T., *et al.* 3D MnO_2-graphene composites with large areal capacitance for high-performance asymmetric supercapacitors. *Nanoscale* **5**, 6790-6796 (2013).

22. Lv P., Zhang P., Feng Y., Li Y., Feng W. High-performance electrochemical capacitors using electrodeposited MnO_2 on carbon nanotube array grown on carbon fabric. *Electrochim. Acta* **78**, 515-523 (2012).

23. Sun P., *et al.* Ultrathin MnO_2 nanoflakes deposited on carbon nanotube networks for symmetrical supercapacitors with enhanced performance. *J. Power Sources* **341**, 27-35 (2017).

24. Huang Z. H., Song Y., Feng D. Y., Sun Z., Sun X., Liu X. X. High mass loading MnO_2 with hierarchical nanostructures for supercapacitors. *ACS Nano* **12**, 3557-3567 (2018).

25. Xu L., Jia M., Li Y., Jin X., Zhang F. High-performance MnO_2-deposited graphene/activated carbon film electrodes for flexible solid-state supercapacitor. *Sci. Rep.* **7**, 12857 (2017).

26. Zhu C., *et al.* Self-branched α-MnO_2/δ-MnO_2 heterojunction nanowires with enhanced pseudocapacitance. *Mater. Horizons* **4**, 451-422 (2017).

27. Chen C., *et al.* All-wood, low tortuosity, aqueous, biodegradable supercapacitors with ultra-high capacitance. *Energy Environ. Sci.* **10**, 538-545 (2017).

28. Shi X., *et al.* Defective carbon nanotube forest grown on stainless steel encapsulated in MnO_2 nanosheets for supercapacitors. *Electrochim. Acta* **278**, 61-71 (2018).

29. Zhang G., Ren L., Hu D., Gu H., Zhang S. Sulfuric acid etching for fabrication of porous MnO_2 for high-performance supercapacitor. *Journal of Colloid Interface Science* **518**, 84-91 (2018).

30. Ye Z., Li T., Ma G., Peng X., Zhao J. Morphology controlled MnO_2 electrodeposited on carbon fiber paper for high-performance supercapacitors. *J. Power Sources* **351**, 51-57 (2017).

31. Pan Z., *et al.* High electroactive material loading on a carbon nanotube@3D graphene aerogel for high-performance flexible all-solid-state asymmetric supercapacitors. *Adv. Funct. Mater.* **27**, 1701122 (2017).

32. Qian J., *et al.* Aqueous manganese dioxide ink for paper-based capacitive energy storage devices. *Angew. Chem. Int. Ed.* **54**, 6800-6803 (2015).

33. El-Kady M. F., *et al.* Engineering three-dimensional hybrid supercapacitors and microsupercapacitors for high-performance integrated energy storage. *Proc. Natl. Acad. Sci. U.S.A.* **112**, 4233-4238 (2015).

34. Zhu S., *et al.* Structural directed growth of ultrathin parallel birnessite on β-MnO_2 for high-performance asymmetric supercapacitors. *ACS Nano* **12**, 1033-1042 (2018).

35. Feng D.-Y., Sun Z., Huang Z.-H., Cai X., Song Y., Liu X.-X. Highly loaded manganese oxide with high rate capability for capacitive applications. *J. Power Sources* **396**, 238-245 (2018).

36. Kang J., *et al.* Electroplated thick manganese oxide films with ultrahigh capacitance. *Adv. Energy Mater.* **3**, 857-863 (2013).

37. Xu H., Hu X., Yang H., Sun Y., Hu C., Huang Y. Flexible asymmetric micro-supercapacitors based on Bi_2O_3 and MnO_2 nanoflowers: larger areal mass promises higher energy density. *Adv. Energy Mater.* **5**, 1401882 (2015).

38. Brown B., Cordova I. A., Parker C. B., Stoner B. R., Glass J. T. Optimization of active manganese oxide electrodeposits using graphenated carbon nanotube electrodes for supercapacitors. *Chem. Mater.* **27**, 2430-2438 (2015).

39. Xu C., *et al.* An ultralong, highly oriented nickel-nanowire-array electrode scaffold for high-performance compressible pseudocapacitors. *Adv. Mater.* **28**, 4105-4110 (2016).

40. Liu Y., Zeng Z., Bloom B., Waldeck D. H., Wei J. Stable low-current electrodeposition of α-MnO_2 on superaligned electrospun carbon nanofibers for high-performance energy storage. *Small* **14**, (2018).

41. Song Y., Feng D.-Y., Liu T., Li Y., Liu X.-X. Controlled partial-exfoliation of graphite foil and integration with MnO_2 nanosheets for electrochemical capacitors *Nanoscale* **7**, 3581-3587 (2015).

42. Du L., Yang P., Yu X., Liu P., Song J., Mai W. Flexible supercapacitors based on carbon nanotube/MnO_2 nanotube hybrid porous films for wearable electronic devices *J. Mater. Chem. A* **2**, 17651-17567 (2014).

43. Gao Y., *et al.* Highly flexible and transferable supercapacitors with ordered three-dimensional MnO_2/Au/MnO_2 nanospike arrays. *J. Mater. Chem. A* **3**, 10199-10204 (2015).

44. Hou Y., Cheng Y., Hobson T., Liu J. Design and synthesis of hierarchical MnO_2 nanospheres/carbon nanotubes/conducting polymer ternary composite for high performance electrochemical electrodes. *Nano Lett.* **10**, 2727-2733 (2010).

45. Chen W., Rakhi R. B., Hu L., Xie X., Cui Y., Alshareef H. N. High-performance nanostructured supercapacitors on a sponge. *Nano Lett.* **11**, 5165-5172 (2011).

46. Fischer A. E., Pettigrew K. A., Rolison D. R., Stroud R. M., Long J. W. Incorporation of homogeneous, nanoscale MnO_2 within ultraporous carbon structures via self-limiting electroless deposition: implications for electrochemical capacitors. *Nano Lett.* **7**, 281-286 (2006).

47. Lee T. H., Pham D. T., Sahoo R., Seok J., Luu T. H. T., Lee Y. H. High energy density and enhanced stability of asymmetric supercapacitors with mesoporous MnO_2@CNT and nanodot MoO_3@CNT free-standing films. *Energy Storage Mater.* **12**, 223-231 (2018).

48. Shi K., Giapis K. P. Scalable fabrication of supercapacitors by nozzle-free electrospinning. *ACS Appl. Energy Mater.* **1**, 296-300 (2018).

49. Meng X., Lu L., Sun C. Green synthesis of three-dimensional MnO_2/graphene hydrogel composites as a high-performance electrode material for supercapacitors. *ACS Appl. Mater. Interfaces* **10**, 16474-16481 (2018).

50. Saito Y., *et al.* Manganese dioxide nanowires on carbon nanofiber frameworks for efficient electrochemical device electrodes. *RSC Adv.* **7**, 12351-12358 (2017).

51. Sumboja A., Foo C. Y., Wang X., Lee P. S. Large areal mass, flexible and free-standing reduced graphene oxide/manganese dioxide paper for asymmetric supercapacitor device. *Adv. Mater.* **25**, 2809-2815 (2013).

52. Liu M., Gan L., Xiong W., Xu Z., Zhu D., Chen L. Development of MnO_2/porous carbon microspheres with a partially graphitic structure for high performance supercapacitor electrodes. *J. Mater. Chem. A* **2**, 2555-2562 (2014).

53. Shi K., Zhitomirsky I. Asymmetric supercapacitors based on activated-carbon-coated carbon nanotubes. *ChemElectroChem* **2**, 396-403 (2015).

54. Lytle J. C.*, et al.* The right kind of interior for multifunctional electrode architectures: carbon nanofoam papers with aperiodic submicrometre pore networks interconnected in 3D. *Energy Environ. Sci.* **4**, 1913-1925 (2011).
